# Nanoencapsulation Enhances the Post-Emergence Herbicidal Activity of Atrazine against Mustard Plants

**DOI:** 10.1371/journal.pone.0132971

**Published:** 2015-07-17

**Authors:** Halley Caixeta Oliveira, Renata Stolf-Moreira, Cláudia Bueno Reis Martinez, Renato Grillo, Marcelo Bispo de Jesus, Leonardo Fernandes Fraceto

**Affiliations:** 1 Department of Animal and Plant Biology, UEL–University of Londrina, Londrina, Paraná, Brazil; 2 Department of Physiological Sciences, UEL–University of Londrina, Londrina, Paraná, Brazil; 3 Department of Biochemistry, Institute of Biology, UNICAMP—University of Campinas, Campinas, São Paulo, Brazil; 4 Department of Environmental Engineering, UNESP–Universidade Estadual Paulista, Sorocaba, São Paulo, Brazil; 5 Nano-Cell Interactions Laboratory, Institute of Biology, UNICAMP—University of Campinas, Campinas, São Paulo, Brazil; National Research Council of Italy, ITALY

## Abstract

Poly(epsilon-caprolactone) (PCL) nanocapsules have been recently developed as a modified release system for atrazine, an herbicide that can have harmful effects in the environment. Here, the post-emergence herbicidal activity of PCL nanocapsules containing atrazine was evaluated using mustard (*Brassica juncea*) as target plant species model. Characterization of atrazine-loaded PCL nanocapsules by nanoparticle tracking analysis indicated a concentration of 7.5 x 10^12^ particles mL^-1^ and an average size distribution of 240.7 nm. The treatment of mustard plants with nanocapsules carrying atrazine at 1 mg mL^-1^ resulted in a decrease of net photosynthesis and PSII maximum quantum yield, and an increase of leaf lipid peroxidation, leading to shoot growth inhibition and the development of severe symptoms. Time course analysis until 72 h after treatments showed that nanoencapsulation of atrazine enhanced the herbicidal activity in comparison with a commercial atrazine formulation. In contrast to the commercial formulation, ten-fold dilution of the atrazine-containing nanocapsules did not compromise the herbicidal activity. No effects were observed when plants were treated with nanocapsules without herbicide compared to control leaves sprayed with water. Overall, these results demonstrated that atrazine-containing PCL nanocapsules provide very effective post-emergence herbicidal activity. More importantly, the use of nanoencapsulated atrazine enables the application of lower dosages of the herbicide, without any loss of efficiency, which could provide environmental benefits.

## Introduction

The growing demand for food production has been accompanied by increased use of herbicides to control weeds and maximize crop productivity. The indiscriminate use of herbicides has been associated with serious harm to the environment due to the contamination of water resources and food, leading to deleterious effects on non-target plants, aquatic species, and sometimes humans [1]. It is therefore essential to develop technologies that minimize the negative impacts caused by these compounds, without hindering crop production.

Nanotechnology, the term used to describe the processes of generation, manipulation, and use of nanomaterials, has emerged as a promising field that could help in alleviating problems associated with agrochemicals [2–7]. Nanomaterials have physicochemical characteristics that differ from those of the same material at the macroscopic scale, with an increase in specific surface area and changes in optical, magnetic, or mechanical properties [8]. There is a great variety of nanomaterials in the form of nanoparticles that can be used as carrier systems for bioactive compounds such as fertilizers and pesticides, with great potential for application in agricultural systems [3–7, 9]. Carrier systems based on nanoparticles provide modified release of bioactive agents, extending their duration of action and directing them more efficiently to target organisms [3]. In the case of herbicides, the use of nanoparticles could be beneficial in many respects, such as decreased contamination of water resources by leaching and less risk of harming non-target organisms, including humans [2,5,6,10].

Atrazine (6-chloro-N2-ethyl-N4-isopropyl-1,3,5-triazine-2,4-diamine) is an herbicide widely used for pre- and post-emergence control of weeds in maize, sorghum, and sugarcane cultures [11]. In common with other triazine herbicides, atrazine acts as an inhibitor of photosynthesis by specific binding to photosystem II, interrupting acyclic electron transport, and decreasing ATP and NADPH synthesis and CO_2_ assimilation [12]. The blockage of photosynthetic electron flow may additionally result in the generation of high amounts of reactive oxygen species (ROS). If ROS production overwhelms antioxidant and photoprotective mechanisms, it causes oxidative stress with consequent damage to proteins, lipids, and other cell components [12]. Macroscopically, atrazine induces symptoms such as leaf chlorosis and growth inhibition, which may lead to leaf necrosis and plant death [11].

Due to its slow degradation under natural conditions, the intensive use of atrazine has been associated with the contamination of soils and water resources [13,14]. In particular, studies have demonstrated the negative effects of atrazine on the metabolism and development of animal and plant species in aquatic ecosystems [15–17]. Additionally, the high persistence of this herbicide in soils may lead to negative effects on the growth of non-target plants, including crops cultivated in the years following applications, as well as native plant species in adjacent preservation areas [15]. Atrazine accumulation is also harmful to human health, causing dysfunctions in the reproductive system and possible tumor development [18].

Recently, we developed atrazine carrier systems based on nanoparticles as a promising strategy to minimize the contamination of natural resources by this compound [19–21]. Polymeric nanocapsules were prepared with poly(epsilon-caprolactone) (PCL), a biodegradable aliphatic polyester that is nontoxic to humans and the environment [22]. *In vitro* assays demonstrated high colloidal stability and a good efficiency of encapsulation of atrazine in the PCL nanocapsules, as well as a modified release profile [20,21]. Interestingly, genotoxicity tests (using plant and human cells) and cytogenetic experiments (using human cells) showed that PCL nanocapsules containing atrazine were less toxic, compared with the free herbicide [20,21,23]. Similar results were obtained in ecotoxicological assays using the alga *Pseudokirchneriella subcapitata* [23]. Additionally, the use of nanocapsules decreased the mobility of atrazine in the soil and provided greater pre-emergence herbicidal activity against mustard seedlings, compared with a commercial atrazine formulation [21]. These results suggested that PCL nanocapsules containing atrazine have great potential for application in agriculture. However, new studies are necessary in order to achieve a deeper characterization of the herbicidal activity of these atrazine carrier systems, as well as to determine the effectiveness of the nanocapsules when applied after emergence of the target plants.

The aim of this study was to evaluate the post-emergence herbicidal activity of PCL nanocapsules carrying atrazine, using mustard plants as a target species model. Firstly, atrazine-containing PCL nanocapsules were further characterized in terms of their concentration and size distribution. The effects of the formulations on the growth and physiological and biochemical parameters of mustard plants were then evaluated and compared with the effects induced by a commercial atrazine formulation.

## Materials and Methods

### Chemicals

Poly(epsilon-caprolactone) - PCL, atrazine, polysorbate 80 (Tween 80), sorbitan monostearate surfactant (Span 60), and xylenol orange were purchased from Sigma-Aldrich. Thiobarbituric acid was obtained from MP Biomedicals. A commercial atrazine formulation (Gesaprim 500 CG) was obtained from Syngenta. Other reagents (analytical grade or better) were purchased from local suppliers.

### Preparation of poly(epsilon-caprolactone) nanocapsules

Nanocapsules were prepared following the protocol described by [20]. The method essentially consisted of mixing an organic phase into an aqueous phase. The organic phase was composed of 100 mg of polymer (PCL), 30 mL of organic solvent (acetone), 200 mg of oil (triglycerides of capric and caprylic acids, in the form of Myritol 318), 40 mg of sorbitan monostearate surfactant (Span 60), and 10 mg of atrazine. The aqueous phase was composed of 30 mL of a solution containing 60 mg of polysorbate 80 surfactant (Tween 80). After dissolving the components of both phases, the organic phase was slowly inserted into the aqueous phase, with magnetic stirring. The resulting suspension was maintained under agitation for 10 min, after which the organic solvent was evaporated under reduced pressure using a rotary evaporator. The atrazine concentration was 1 mg mL^-1^. Nanocapsules prepared without atrazine were used as controls in the experiments.

### Size measurement by nanoparticle tracking analysis (NTA)

The concentrations and size distributions of the PCL nanocapsules (with or without atrazine) were determined using a NanoSight LM 10 cell (Malvern Instruments, UK) with a green laser (532 nm) and a high specification CMOS camera controlled using NanoSight v. 2.3 software. The nanocapsule suspensions were diluted 5,000 times and the samples were analyzed using five measurements, with approximately 4,000 tracks counted in each analysis. Each sample replicate was injected into the volumetric cell (1 mL), displacing the sample that had been measured previously. The data were analyzed using GraphPad Prism 6 software and all the experiments were performed in pentaplicate (n = 5).

### Plant material and growth conditions

*Brassica juncea* (L.) Czern. (Florida broad leaf mustard) was used as the target species model. Seeds were purchased from Isla Sementes (Porto Alegre, Brazil). Germination was performed in plastic pots (10.5 cm high, 9.5 cm lower diameter, 14 cm upper diameter) filled with a clay soil (a Rhodic Ferralsol) collected from an herbicide-free experimental area at the campus of the State University of Londrina, Brazil (for a detailed characterization of the soil, see [24]). One week after sowing, four individuals were retained in each pot and the substrate was supplemented with 50 mL of complete nutrient solution [25]. Throughout the cultivation, the plants were kept in a greenhouse under natural conditions of light, relative humidity, and temperature. The experiments were carried out from June to September (winter). The average daily values and standard deviations of temperature, relative humidity, and accumulated global solar radiation were 18.9 ± 3.1 °C, 78.2 ± 13.6%, and 11.9 ± 4.5 MJ m^-2^, respectively (data kindly provided by the Laboratory of Agrometeorology, Embrapa Soja, Londrina).

### Herbicidal activity conditions

Mustard plants aged 30 days were used for post-emergence treatments with the following samples: distilled water (control), nanocapsules without atrazine (NC), commercially formulated atrazine (ATZ), and nanocapsules containing atrazine (NC+ATZ). The standard concentration of atrazine in the nanoformulation was 1 mg mL^-1^. Each pot (152 cm^2^ surface area) containing four plants was sprayed with 3.1 mL of the test sample, resulting in application of the atrazine dosage recommended by the manufacturer (2000 g atrazine per hectare). Nanoformulations diluted ten-fold in water (atrazine concentration of 0.1 mg mL^-1^) were also used in order to test the effectiveness of a lower dosage (200 g atrazine per hectare). The treatments were applied in the morning (before 09:00 am).

### Physiological and biochemical characterization

#### Symptom evolution and weight analysis

Macroscopic symptoms in the leaves were recorded 3 and 7 days after the treatments, using a Samsung ST200F camera. The images were taken under the same conditions, and the white background was used to adjust white balance using the Photoshop CS6 software. For weight analysis, shoots were harvested 7 days after treatment and kept for 72 h at 60 °C, prior to dry weight measurement.

#### Chlorophyll fluorescence

Chlorophyll *a* fluorescence parameters were measured before dawn using an OS1p fluorometer (Opti-Sciences, Hudson, NH, USA). The measurements were carried out on the adaxial surface of dark-adapted leaves; the maximal fluorescence (F_m_) was measured after applying a 0.8 s saturating irradiance pulse (8,250 μmol m^-2^ s^-1^). The maximum quantum yield of the photosystem II photochemistry was expressed as F_v_/F_m_ = (F_m_−F_0_)/F_m_ [26].

#### Leaf gas exchange

Leaf gas exchange parameters (net photosynthesis, stomatal conductance, intercellular CO_2_ concentration, and transpiration) were measured using a Portable Photosynthesis System (LI-6400XT, LI-COR Biosciences, Lincoln, NE, USA). The infrared gas analyzer (IRGA) was connected to a 6 cm^2^ 6400-02B measuring chamber with LED light source where the leaves were exposed to a saturating PAR (1,000 μmol m^-2^ s^-1^). All the measurements were carried out on sunny days, between 08:00 and 10:00 am.

#### Oxidative stress

Hydrogen peroxide and lipid peroxidation were analyzed as markers of oxidative stress. Freshly collected leaves (100 mg) were homogenized with cold TCA (0.2%) diluted in methanol. The supernatant obtained after centrifugation at 10,000 x *g* for 5 min was used for the measurements. Hydrogen peroxide was detected spectrophotometrically at 560 nm after its reaction with xylenol orange in the presence of FeSO_4_ in acidified medium [27]. Lipid peroxidation was determined by the analysis of thiobarbituric acid reactive substances (TBARS), following the procedure described by [28].

### Statistical analysis

In each experiment, four pots containing four plants were used per treatment. Sixteen biological replicates were used for weight analysis, nine for gas exchange experiments, and five for chlorophyll fluorescence and oxidative stress analysesThe data were analyzed using two-way ANOVA followed by the Tukey post-hoc test (p < 0.05), except for shoot weight data which were analyzed by one-way ANOVA.

## Results

### Characterization of the nanocapsules containing atrazine

**Fig 1:**
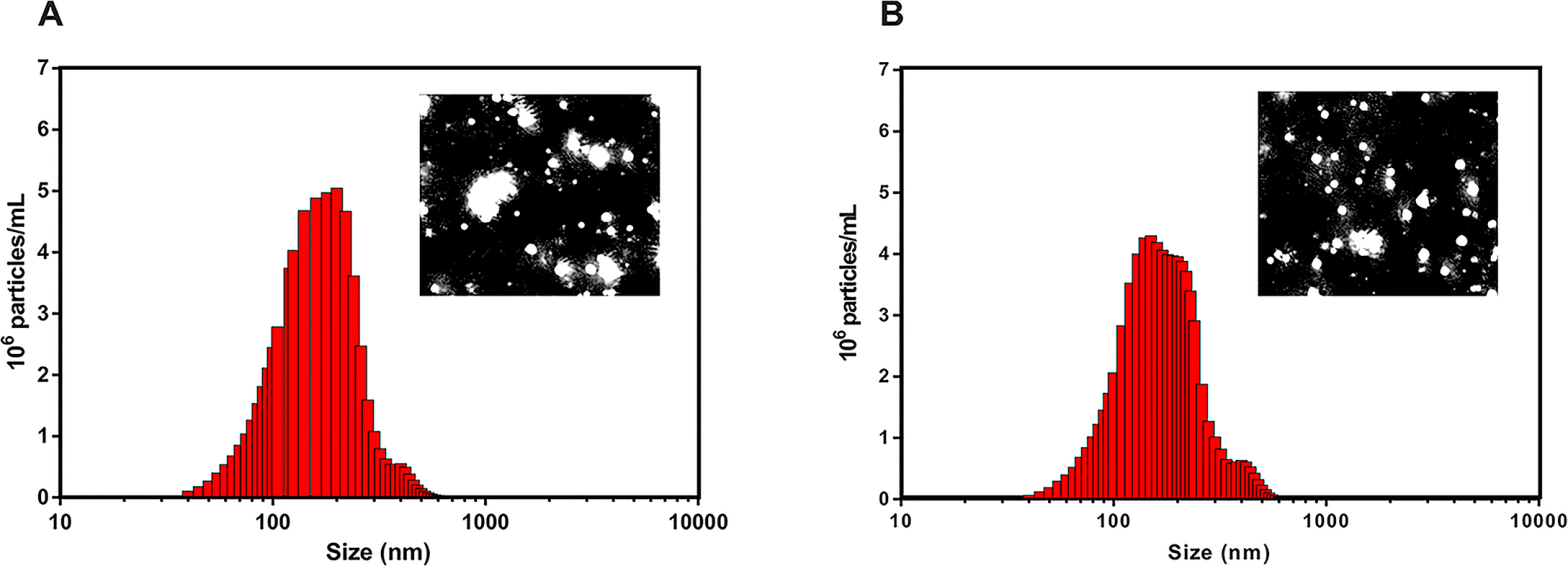
Size distribution of PCL nanocapsules. Size distribution of PCL nanocapsules from NTA measurements are shown with the corresponding NTA video frame: (A) without atrazine and (B) with atrazine. The NP suspensions were diluted 5,000 times, and the samples were analyzed at 25 °C using five measurements, with approximately 4,000 tracks counted in each analysis.

The encapsulation efficiency, colloidal stability and physicochemical characteristics of the polymeric nanocapsules (with and without atrazine) have been described previously [20]. However, a complementary study using nanoparticle tracking analysis (NTA) was performed in order to determine the concentration and size distribution of the nanocapsules used in this study. Direct real-time observation of the Brownian motion of the nanocapsules showed that the concentration of nanocapsules in suspension (with and without atrazine) was approximately 7.5 x 10^12^ particles mL^-1^ (Fig 1). The average size distributions of the PCL nanocapsules (± standard error) were 221.0 ± 2.5 nm (without atrazine, Fig 1A) and 240.7 ± 2.9 nm (with atrazine, Fig 1B), which are in good agreement with the values obtained previously using dynamic light scattering and microscopy analysis [20].

### Herbicidal activity assays

#### Symptom evolution and weight analysis

**Fig 2:**
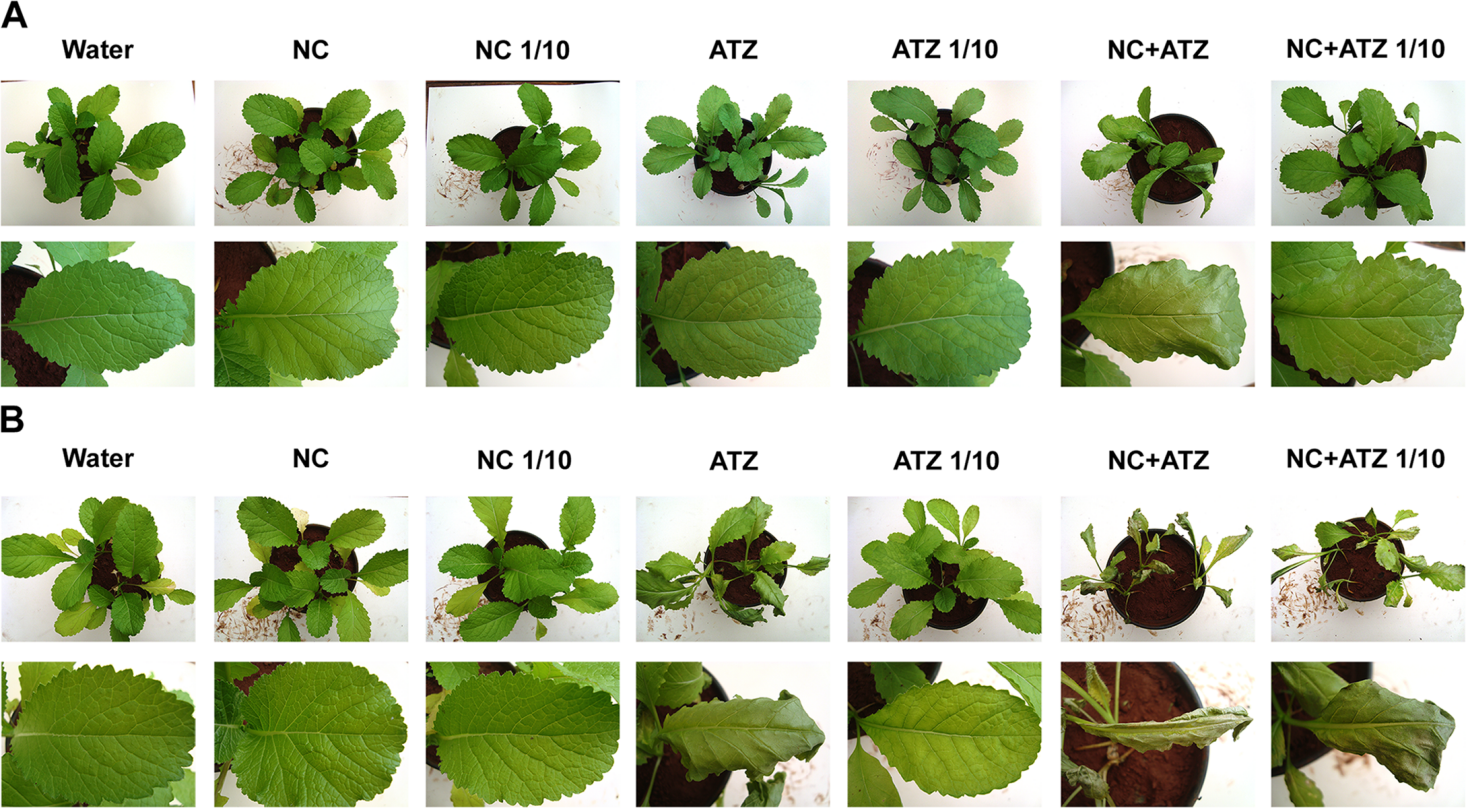
Symptom evolution in *B*. *juncea* leaves treated with the formulations. Symptoms were recorded (A) 3 and (B) 7 days after the plants were sprayed with 3.1 mL of water, empty PCL nanocapsules (NC), commercial atrazine (ATZ), or PCL nanocapsules containing atrazine (NC+ATZ). The formulations containing atrazine at 1 mg mL^-1^ were used undiluted or after 10-fold dilution in water (1/10), resulting in atrazine dosages equivalent to 2000 or 200 g ha^-1^, respectively.

The first macroscopic symptoms of atrazine toxicity were observed 3 days after the treatments, but only in leaves that were sprayed with nanocapsules containing atrazine (Fig 2A). After 7 days, the leaves of the plants treated with nanocapsules containing atrazine at the recommended concentration were mostly necrotic and wilted, with a chlorotic petiole (Fig 2B). A comparison of the treatment with ten-fold diluted nanocapsules containing atrazine (NC+ATZ 1/10) with that using commercial atrazine at the recommended dosage (ATZ) revealed similar symptoms of leaf wilt, yellowing, and necrosis. However, treatment with the diluted commercial atrazine resulted in much milder symptoms, which were limited to leaf chlorosis.

**Fig 3:**
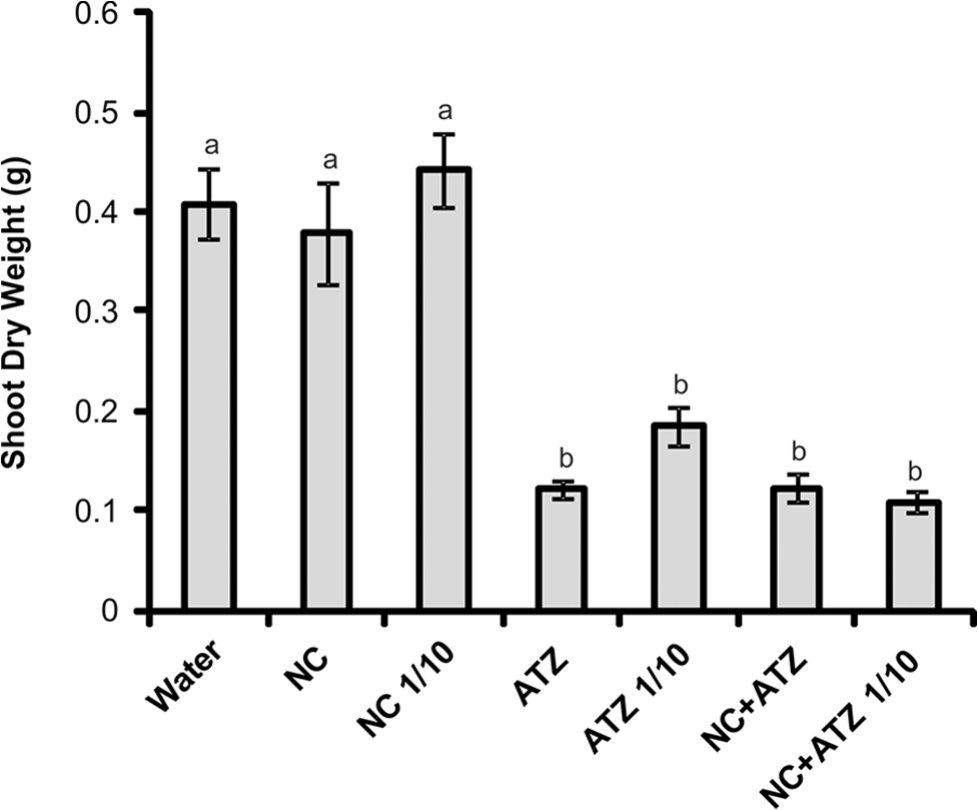
Shoot dry weights of *B*. *juncea* plants treated with the formulations. Shoots were sampled 7 days after the plants were sprayed with 3.1 mL of water, empty PCL nanocapsules (NC), commercial atrazine (ATZ), or PCL nanocapsules containing atrazine (NC+ATZ). The formulations containing atrazine at 1 mg mL^-1^ were used undiluted or after 10-fold dilution in water (1/10), resulting in atrazine dosages equivalent to 2000 or 200 g ha^-1^, respectively. Different letters indicate significantly different values according to one-way ANOVA followed by the Tukey test (p < 0.05). Data are shown as means ± SE (n = 16).

All the treatments containing atrazine resulted in strong reductions of shoot dry weight, compared with plants sprayed with water (Fig 3). Although not statistically significant, a 50% higher shoot dry weight was obtained for the plants sprayed with commercial atrazine at 10% of the recommended dosage, suggesting that dilution resulted in reduced effectiveness of this formulation. On the other hand, the same dilution of nanocapsules containing atrazine did not affect the shoot dry weight reduction caused by the herbicide.

In the case of the plants sprayed with nanocapsules without atrazine, no macroscopic symptoms were observed after 3 and 7 days, irrespective of the level of dilution (Fig 2A and 2B). The dry weight of mustard plant shoots was also not affected by the treatment with empty nanocapsules (Fig 3). These results suggest that the nanocapsules alone do not influence the shoot growth and appearance of the plants.

#### Physiological parameters

**Fig 4:**
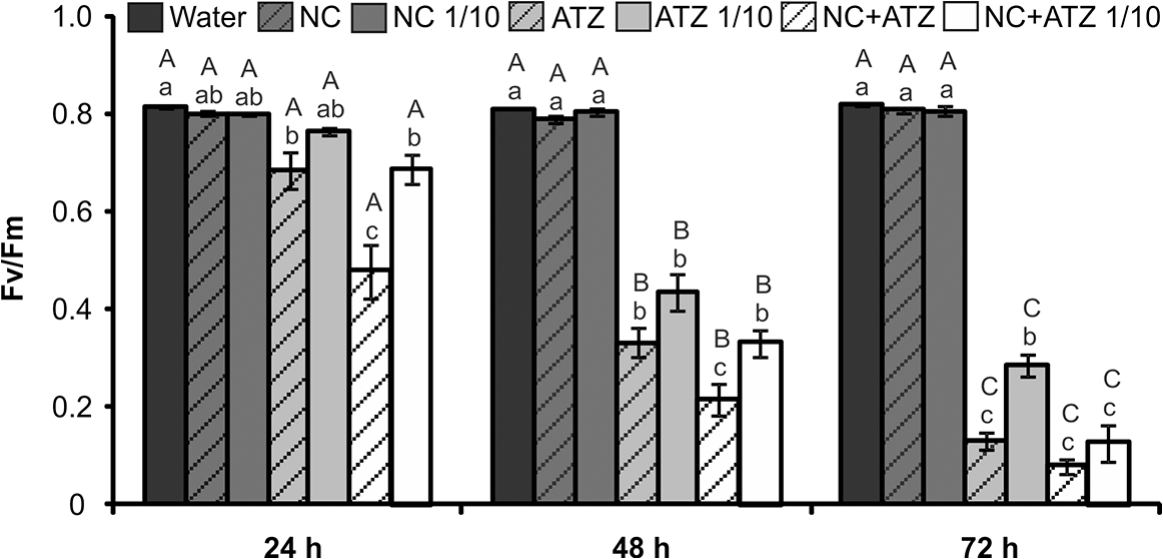
Maximum photosystem II quantum yields of *B*. *juncea* plants treated with the formulations. Chlorophyll fluorescence parameters were evaluated 24, 48, and 72 h after the plants were sprayed with 3.1 mL of water, empty PCL nanocapsules (NC), commercial atrazine (ATZ), or PCL nanocapsules containing atrazine (NC+ATZ). The formulations containing atrazine at 1 mg mL^-1^ were used undiluted or after 10-fold dilution in water (1/10), resulting in atrazine dosages equivalent to 2000 or 200 g ha^-1^, respectively. Different letters indicate significantly different values according to two-way ANOVA followed by the Tukey test (p < 0.05). Lowercase letters indicate comparison among all treatments at each time point, while capital letters indicate comparison of the same treatment along its individual time course analysis. Data are shown as means ± SE (n = 5).

Time course analysis of the F_v_/F_m_ ratio over 72 h showed that application of the atrazine-containing formulations to the plants led to a gradual decrease in the maximum quantum yield of photosystem II (PSII) (Fig 4). After 24 and 48 h, the inhibition of PSII photochemistry was more intense in leaves treated with undiluted nanocapsules carrying atrazine. For all the time points analyzed, similar decreases in the F_v_/F_m_ ratio were observed for the treatments using commercial atrazine at 1 mg mL^-1^ and the nanocapsules containing atrazine at 0.1 mg mL^-1^. At no time did the F_v_/F_m_ ratio of leaves treated with nanocapsules without atrazine differ from that of control leaves sprayed with water.

**Fig 5:**
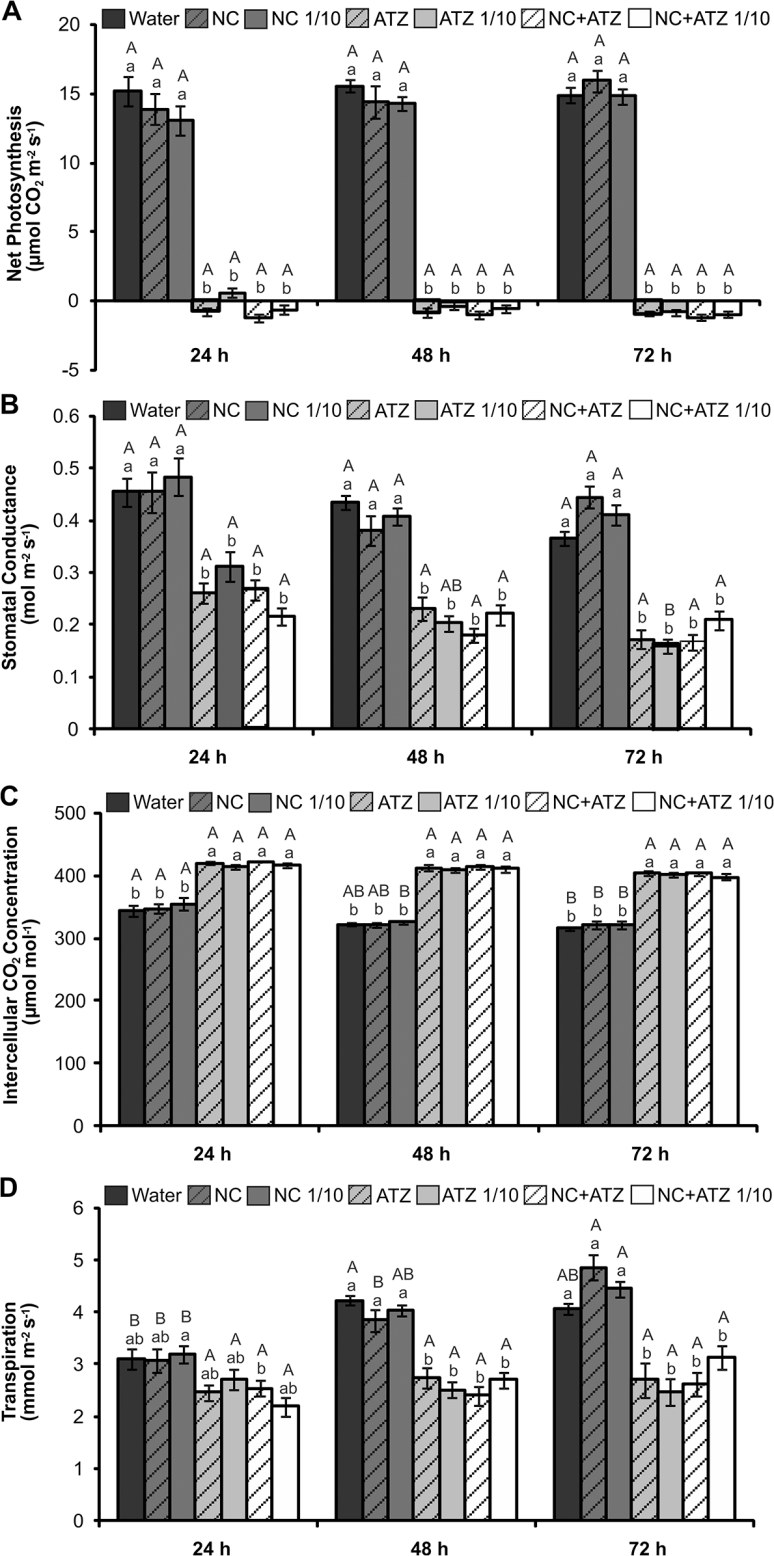
Leaf gas exchange parameters of *B*. *juncea* plants treated with the formulations. Net photosynthesis (A), stomatal conductance (B), intercellular CO_2_ concentration (C), and transpiration (D) were evaluated 24, 48, and 72 h after the plants were sprayed with 3.1 mL of water, empty PCL nanocapsules (NC), commercial atrazine (ATZ), or PCL nanocapsules containing atrazine (NC+ATZ). The formulations containing atrazine at 1 mg mL^-1^ were used undiluted or after 10-fold dilution in water (1/10), resulting in atrazine dosages equivalent to 2000 or 200 g ha^-1^, respectively. Different letters indicate significantly different values according to two-way ANOVA followed by the Tukey test (p < 0.05). Lowercase letters indicate comparison among all treatments at each time point, while capital letters indicate comparison of the same treatment along its individual time course analysis. Data are shown as means ± SE (n = 9).

The leaf gas exchange parameters were also affected by atrazine (Fig 5). From 24 h following treatment, all the atrazine-containing formulations caused drastic decreases in the net photosynthesis rate (Fig 5A), as well as decreases in stomatal conductance (Fig 5B) and increases in leaf intercellular CO_2_ concentrations (Fig 5C). The transpiration rate was negatively affected by these formulations from 48 h following treatment (Fig 5D). It is noteworthy that at 24 h after treatment with nanocapsules carrying atrazine (regardless of the dosage), the leaves showed negative photosynthetic rates (Fig 5A), indicating that carbon assimilation by photosynthesis was overwhelmed by respiration. In the case of commercial atrazine at the lower concentration tested, this negative carbon balance was only obtained 48 h after the treatment (Fig 5A), as well as the rate of decrease of stomatal conductance was slower than that of other atrazine-containing formulations (Fig 5B). The treatment using empty nanocapsules did not affect any of the gas exchange parameters when compared to control leaves at the same time point.

#### Oxidative stress parameters

**Fig 6:**
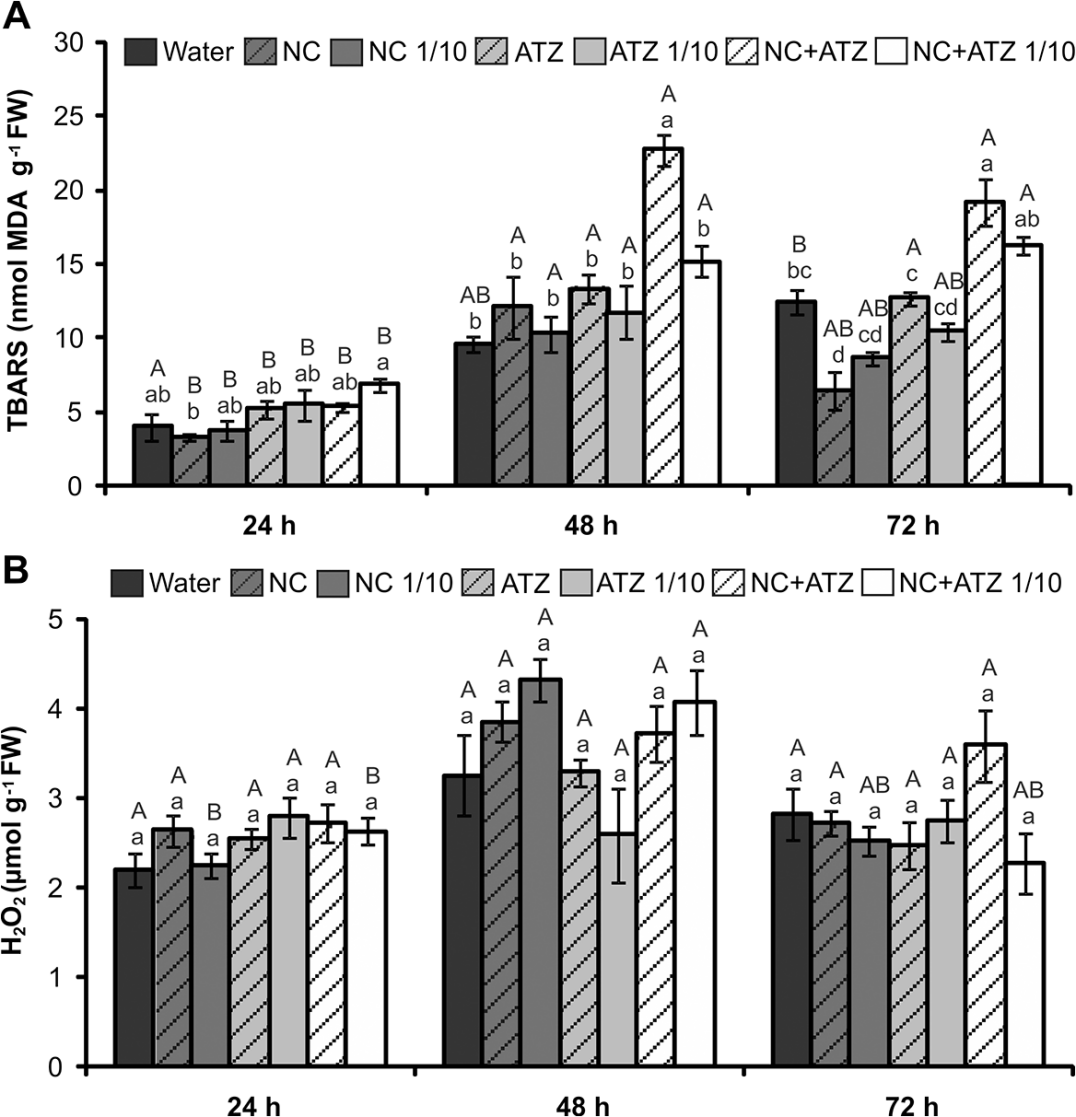
Oxidative stress parameters of *B*. *juncea* plants treated with the formulations. Lipid peroxidation (A) and hydrogen peroxide content (B) were evaluated 24, 48, and 72 h after the plants were sprayed with 3.1 mL of water, empty PCL nanocapsules (NC), commercial atrazine (ATZ), or PCL nanocapsules containing atrazine (NC+ATZ). The formulations containing atrazine at 1 mg mL^-1^ were used undiluted or after 10-fold dilution in water (1/10), resulting in atrazine dosages equivalent to 2000 or 200 g ha^-1^, respectively. Different letters indicate significantly different values according to two-way ANOVA followed by the Tukey test (p < 0.05). Lowercase letters indicate comparison among all treatments at each time point, while capital letters indicate comparison of the same treatment along its individual time course analysis. Data are shown as means ± SE (n = 5).

Time course analysis indicated an overall enhancement of leaf lipid peroxidation in most treatments during the experiment (Fig 6A). However, 48 h after treatment, higher MDA content was detected in leaves sprayed with the undiluted formulation of nanocapsules containing atrazine, compared with the control plants (Fig 6A). Regardless of the applied concentration, at 72 h the leaves treated with encapsulated atrazine presented higher lipid peroxidation than those sprayed with the commercial atrazine formulations (Fig 6A). No significant changes in hydrogen peroxide levels were detected in leaves treated with any of the formulations in comparison with water-sprayed leaves at the same time point (Fig 6B). Individual time course analysis showed only a transient increase of hydrogen peroxide content at 48 h in leaves treated with NC 1/10 or NC+ATZ 1/10 (Fig 6B).

## Discussion

The development of modified release systems for agrochemicals has increased in recent years, together with the need to evaluate the effectiveness of these systems in controlling target species [7]. However, there have been only a limited number of studies concerning the performance of pesticides in modified release systems produced using polymeric nanocarriers [6,21,29–31]. Moreover, studies analyzing the effect of nanopesticides on plant physiology are still lacking in the literature. Our previous work has suggested that PCL nanocapsules containing atrazine could be potentially useful in agriculture, because the implementation of such systems could reduce the harm caused by this herbicide to the environment and human health [20,21]. In the present study, these atrazine-loaded PCL nanocapsules were further characterized by nanoparticle tracking analysis. The post-emergence herbicidal activities of different formulations were then evaluated by means of a detailed analysis of their effects on biochemical, physiological, and growth parameters of mustard plants, including comparison with a commercially available atrazine product.

The phytotoxicity assays demonstrated that encapsulation not only maintained the mechanism of action of atrazine, but also potentiated its herbicidal activity against mustard plants when compared with the effects of the commercial atrazine product. Thus, it was possible to reduce the atrazine dosage ten-fold, without compromising the biological activity of the herbicide. This conclusion was supported by various results obtained in the present study. When the formulations were applied at the recommended atrazine dosage (2000 g ha^-1^), the nanocapsules containing atrazine induced faster and more severe development of symptoms (Fig 2), faster inhibition of PSII photochemistry (Fig 4), and greater lipid peroxidation (Fig 6) in mustard leaves, compared with the commercial atrazine product. Moreover, the use of the ten-fold diluted encapsulated atrazine formulation resulted in very similar (Figs 2B, 3, 4, and 5) or more intense (Figs 2A and 6A) effects on the analyzed parameters of the mustard plants, compared with the use of the commercial herbicide at the standard dosage. In contrast, the use of a lower concentration of commercial atrazine compromised its herbicidal activity, especially considering the much milder toxicity symptoms in leaves (Fig 2), as well as reduced shoot growth inhibition, compared with the other atrazine-containing formulations (Fig 3).

Several recent studies have shown that nanoformulations can maintain or enhance the biological activity of pesticides against target species (reviewed by [7]). Kumar and colleagues [30] showed that imidacloprid encapsulated in sodium alginate nanoparticles was more effective than the active compound alone against sucking pests (leafhopper) in Okra (bhindi) crops. In studies with different plant species, similar results have been reported for herbicides loaded in solid lipid nanoparticles [6], chitosan/tripolyphosphate nanoparticles [29], and silver nanoparticles-chitosan [31]. The atrazine-containing PCL nanocapsules employed here were previously shown to be more effective in controlling mustard plants, compared with the commercial herbicide formulation, when applied before emergence of the seedlings [21]. The results obtained in the present study further demonstrated their effectiveness, in this case using post-emergence application.

Although the findings provided clear evidence of the ability of PCL nanocapsules to increase the activity of atrazine towards a target species, the mechanisms involved remain unclear. As previously suggested [21], the increased herbicidal activity of PCL nanocapsules containing atrazine might be partially explained by the ability of encapsulation to protect the active compound against physicochemical degradation. In the present case, it could be hypothesized that the hydrophobic nanocapsules might interact with the leaf cuticle, hence increasing the delivery of atrazine to the plant tissues, while at the same time decreasing the loss of the herbicide to the environment. Further efforts are required in order to elucidate the precise mechanisms by which PCL nanocapsules increase the herbicidal activity of atrazine. New studies are in progress, involving the labeling of nanoparticles with a fluorescent probe in order to investigate their possible interactions with leaves.

The enhanced herbicidal activity obtained by atrazine nanoencapsulation might also be associated with the modified release of the herbicide by PCL nanocapsules, since it would promote the gradual contact between the herbicide and the plant. In previous study [20], we have described the release profile of atrazine-containing PCL nanocapsules and it was observed that the required time for 50% release (t_50%_) was approximately 30 hours, while t_50%_ of free atrazine was less than five hours [6,21]. This slow release of atrazine from PCL nanocapsules could be explained by the polymer swelling and relaxation of the polymeric matrix and by the high encapsulation efficiency (86%) [20]. Moreover, NTA analysis indicated good reproducibility and quality of the nanoformulations, bringing further insight into the potential application of PCL nanocapsules in agriculture.

Overall, it can be concluded that PCL nanocapsules provide an efficient carrier system for atrazine and could be used as an effective tool in the post-emergence control of weeds. Due to the enhancement of herbicidal activity in comparison with a commercial formulation, the use of encapsulated atrazine would permit the application of lower dosages of the herbicide. The lower input of herbicides in the environment is a highly desirable feature of sustainable agriculture. Furthermore, the encapsulation of atrazine reduces its mobility in the soil, thereby helping to avoid contamination of the water table due to leaching [21], and also decreases the cytotoxic and genotoxic effects of the herbicide [20,21,23]. The use of PCL nanocapsules could potentially reduce the harm caused to the environment and human health by atrazine, without compromising herbicidal effectiveness.
